# *Crataegus mexicana* root-based supplement intoxication in a woman with weight loss persistent preoccupation: Case report

**DOI:** 10.7705/biomedica.7794

**Published:** 2026-03-02

**Authors:** Sebastián Abad-Cuenca

**Affiliations:** 1 Consultorios Médicos de Especialidades "San Miguel", Azogues, Ecuador Consultorios Médicos de Especialidades "San Miguel" Azogues Ecuador

**Keywords:** Crataegus, poisoning, diarrhea, body image, body weight, México, Crataegus, envenenamiento, diarrea, imagen corporal, peso corporal, México

## Abstract

Body image preoccupation is increasingly common, particularly among Latin American women, influenced by societal beauty standards favoring thinness. This often drives individuals to use unregulated weight loss supplements, which can lead to serious health risks. This case reports a rare instance of acute diarrhea resulting from the unsupervised use of a *Crataegus mexicana* root-based supplement, a product frequently marketed for weight loss. A 41-year-old Hispanic woman presented to the emergency department with a 15-day history of daily diarrhea, epigastric pain, and malaise. She reported using the supplement for weight loss. Physical examination revealed moderate dehydration and localized tenderness in the precordial and epigastric regions. The diagnosis was acute diarrhea related to the herbal supplement, with moderate dehydration. The patient received intravenous saline and analgesics and was discharged in stable condition after 48 hours. Follow-up showed no recurrence of symptoms, but persistent body image concerns. This case highlights the dangers of unsupervised herbal supplement use for weight loss and underscores the need for proper medical guidance when using supplements.

## Resumen

La preocupación persistente por la imagen corporal es cada vez más común, particularmente entre las mujeres latinoamericanas, influenciadas por los estándares de belleza de la sociedad que favorecen la delgadez. A menudo, esto lleva a las personas a usar suplementos no regulados para bajar de peso, lo que puede conducir a graves riesgos para la salud.

Se reporta un caso raro de diarrea aguda como resultado del uso no supervisado de un suplemento a base de raíz de *Crataegus mexicana,* un producto frecuentemente comercializado para la pérdida de peso. Una mujer hispana de 41 años acudió al servicio de urgencias con una historia de 15 días de diarrea diaria, dolor epigástrico y malestar general. Informó que usaba el suplemento para perder peso. En el examen físico se encontró deshidratación moderada y dolor localizado en la región precordial y en la epigástrica. Se diagnosticó diarrea aguda asociada con el consumo del suplemento, con deshidratación moderada. La paciente fue tratada con líquidos intravenosos y analgésicos, y fue dada de alta en condiciones estables tras 48 horas. El seguimiento mostró resolución clínica, aunque persistía la preocupación por la imagen corporal. Este caso evidencia los riesgos del uso de suplementos herbales sin control médico, y destaca la necesidad de regulación y orientación profesional.

In Latin America, especially among women, societal ideals of thinness often drive the pursuit of weight loss through unregulated products. According to STEPS Ecuador 2018, 67.4% of adult women in Ecuador have overweight or obesity, with obesity alone affecting 30.9% ^1^. This context favors the widespread, unsupervised use of herbal products for weight loss, including *Crataegus mexicana* (tejocote), a hawthorn species native to México. Consequently, series of highly consequential sequels of women was observed, as a case in point of an increased corporal image dissatisfaction ^2^. Reports have linked the root to gastrointestinal and cardiac side effects, as well as adulteration with *Thevetia peruviana* (yellow oleander), a plant with lethal potential.

This case report has been reported in line with the SCARE Criteria ^3^.

## Ethical considerations

The informed consent of the patient was obtained for the publication of this clinical case.

## Case report

A 41-year-old Hispanic woman, without comorbidities or relevant familiar history, presented to the emergency department referring to present dialy diarrhea, identified as 7 in the Bristol scale and manifested more than four times per day in low quantity for 15 days. Ten hours before, the clinical profile was exacerbated with middle epigastralgia, diarrhea five times, asthenia, malaise and precordial pain. The patient referred regular exercise (CrossFit) on her habits, and she did not mention any diet change but the onset of hawthorn root supplement (table 1) consumption, one piece per day (49 mg of *tejocote* root), due to the desire for a thinner body image. She denies increasing the dose on her own or taking any other drug or supplement. On presentation, her vital signs were normal and a body mass index (BMI) of 27 kg/m^2^; at the physical exam she presented dryness of the oral mucosa, precordial pain and tenderness in the third, fourth and fifth intercostal spaces, epigastrium and mesogastrium.

**Table 1:** Diagnostic approach for acute diarrhea in this patient

Diagnostic category	Evidence
Infectious causes	No fever, no leukocytosis, no travel, no sick contacts
Parasitic causes	No eosinophilia, no contaminated water consumption
Foodborne toxic ingestion	No recent dietary change or food outbreaks
Drug-induced	No medications other than tejocote root
Herbal supplement-related	Onset after tejocote root ingestion; symptoms resolved after withdrawal

An electrocardiogram was performed, which was normal. Lab results showed mild leukocytosis (11.050/μl) without any other abnormality. The patient was diagnosed with costochondritis, acute diarrhea due to an adverse effect of a dietetic supplement and moderate dehydration due to intestinal loss (table 1). She was admitted for observation and treated with intravenous 0.9% saline solution and 60 mg intravenous ketorolac. The patient was subsequently discharged in a stable and asymptomatic condition with oral diclofenac for five days. The herbal supplementation was suspended.

The woman, after six and twelve months, has been stable, asymptomatic without instability signs. Whereas, her psychological follow-up is still showing weight-loss obsession, with emotional lability when her weight is discussed by family or friends. She has not been treated by a psychologist by her own will.

## Discussion

Body weight dissatisfaction is common and more prevalent among Latin American women than in men, as is body distortion, which is highly likely to lead to psychosocial disorders, according to the Brazilian study of Cabral *et al.*^4^. In 2011, in Sonora (México) more than $18 million Mexican pesos (≈1 million USD) were spent on weight loss products in one month by women aged 30 to 45 ^5^. Cheaper options may be more attractive while they are more dangerous due to the lack of healthcare consultation.

Slimming products are sold as a natural supplementation for those who desire to lose weight in an easier and cheaper way. Many of these products may be compounded by plants which, in some instances, do possess slimming properties. Since they should be administered in a specific way and it best is put in the hands of specialists in this field, to receive correct and individualized advice. Moreover, many people are used to thinking that they have no adverse effects or that they may be effective solely, without improving eating habits and physical activity ^2^^,^^6^. *Tejocote* root *(C. mexicana)* has been presented prominently in many weight-loss products (dietary supplements and herbal remedies) sold in naturist stores. Although its geographical distribution is in México, it has been sold in many Latin American countries ^7^.

The hawthorne *tejocote (C. mexicana)* is a Mesoamerican dicotyledonous plant of the Rosaceae family, and its distribution is mainly in México. The plant itself has been used in traditional medicine for congenital heart disease. Its flowers, leaves, roots, and fruit have cardiac glycoside-like effects and antioxidant activity ^8^. Furthermore, it has proven to have properties as antioxidant, hypoglycemic, hypolipidemic, astringent, antispasmodic, cardiotonic, diuretic, hypotensive and anti-atherosclerotic ^8^^,^^9^. The total phenolic and flavonoid contents is higher in the seed, while the highest levels of flavan-3-ol and proanthocyanidins is found in the peel. The fruit of *C. mexicana* is a significant source of flavonoids and organic acids ^9^. Nevertheless, its consumption has been associated with myalgias, dizziness, nausea, vomiting, diarrhea, bradycardia and effects in plasma digoxin concentration ^10^^,^^11^.

Whether the *Crataegus* genus may have many medical benefits, it is supported by studies that just include other species rather than *C. mexicana,* and other structures but the roots. *Crataegus* spp. was studied by Daniele *et al.*^10^, in a systematic review in 2006, who concluded that the use of preparations containing hawthorn leaves with flowers is considerable, well tolerated and can inhibit the phosphodiesterase activity increasing coronary and myocardial circulation, with a few cases of some severe adverse events. In addition, a double-blind, randomized, controlled study suggested that a *Crataegus* spp. extract mixture *(C. pinnatifida* leaves and *Citrus unshiu* peels) is effective and safe for reducing the body fat and weight and improving the lipid and leptin profiles in overweight subjects ^12^. Nevertheless, its unsupervised use may be problematic.

Though *Crataegus* species are well known to have many flavonoids on its structure, the roots of a similar species, *C. pentagyna,* has shown a higher concentration of naringenin-7-O-neohesperidoside ^13^. Naringenin is known to induce laxative effects because it raises the levels of interstitial cells of Cajal markers ((c-Kit and Stem Cell Factor), as well as aquaporin 3 in mice with loperamide-induced constipation ^14^. Daniele *et al.*^10^ have reported just one case of diarrhea induced by *Crataegus* preparations; although another specie *(C. azarolus)* berries extract has demonstrated to protect against diarrhea ^15^. Thus, due to the similarity between *C. pentagyna* and *C. mexicana,* we postulate that, though its intake is generally considered to be safe, inappropriate root-based supplementation may be responsible for acute diarrhea.

In addition, *T. peruviana* contamination implies further risk. Its glycosides -such as thevetin A/B and neriifolin- inhibit the Na+/K+-ATPase pump in cardiac myocytes, leading to intracellular calcium accumulation, arrhythmias, gastrointestinal effects, and potentially death ^16^. Alerts issued by the U.S. Food and Drug Administration (FDA) and the Centers for Disease Control and Prevention (CDC) in 2023 warned about mislabeled "tejocote root" supplements containing this toxic plant ^17^.

This case adds to the limited clinical literature on *C. mexicana* root toxicity in humans. Its diagnostic value lies in the clear temporal association, symptom resolution after withdrawal, and exclusion of other causes. In the context of recent alerts about adulteration with *T. peruviana,* it underscores the need for heightened clinical suspicion and regulatory oversight of herbal supplements by national organisms like the national agency for health regulation, control and surveillance *(Agencia Nacional de Regulación, Control y Vigilancia Sanitaria,* ARCSA) in Ecuador. Furthermore, this case illustrates the inherent challenge of ensuring consistent pharmacological concentrations in unregulated plant-based products.

While most existing studies have evaluated standardized extracts derived from leaves and fruit, the supplement consumed in this case was composed of root material -raising uncertainty regarding the expected active compounds and their bioavailability. This variability complicates both diagnosis and clinical decision-making.

This report contributes to the ongoing discussion on the need for strict regulation, pharmacovigilance, and research on the adverse effects of over-the-counter botanical supplements in non-standardized dosages. Importantly, it highlights the difficulty of guaranteeing consistent compound concentrations in unregulated plant-based supplements. While this case involved ingestion of root extract (and an apparent adulteration), previous studies have assessed combinations of leaves and fruit, making comparison and prediction of toxicity unreliable.

## Conclusions

Weight loss obsession is common among Latin American women, who are very likely to purchase anything to achieve their desired body image. This case highlights the clinical and regulatory challenges posed by herbal supplements, especially those lacking standardization. *Crataegus mexicana* root, while natural, can induce gastrointestinal and cardiac side effects. Moreover, the risk of adulteration with highly toxic species such as *T. peruviana* requires vigilant regulatory oversight. Clinicians must actively inquire about herbal supplement use in patients with compatible clinical presentations.
